# Vengeful Vines: Cell Death and Neoplasm Formation in Aristolochia (Aristolochiaceae, Piperales) in Response to Oviposition of Troidini (Papilionidae, Lepidoptera) Butterflies

**DOI:** 10.1002/ece3.74426

**Published:** 2026-09-29

**Authors:** Veerle van Beeck, Justinah Parantis, Berber E. Zandstra, Tjomme Fernhout, Arthur Chung, Nazirah Mustaffa, Nina E. Fatouros, Corné F. H. van der Linden

**Affiliations:** ^1^ Biosystematics Group Wageningen University & Research Wageningen the Netherlands; ^2^ Research and Education Division Poring Butterfly Farm Sabah Parks Kota Kinabalu Sabah Malaysia; ^3^ Sabah Forestry Department Forest Research Centre Sandakan Sabah Malaysia; ^4^ Institute for Tropical Biology & Conservation University Malaysia Sabah Kota Kinabalu Malaysia; ^5^ Naturalis Biodiversity Center Leiden the Netherlands

**Keywords:** *Aristolochia*, coevolution, egg killing traits, HR‐like cell death, neoplasm formation, Troidini

## Abstract

The ecological interactions of the iconic Troidini swallowtail butterflies and their host plants remain largely unknown, despite the popularity of these butterflies in collections and their significance as conservation flagships. We sought to elucidate the ecological interactions of perhaps the least known life‐stage of the butterflies: that of the egg with its host plant. We recorded oviposition location and egg survival rates for *Troides helena, Troides amphrysus* and *Pachliopta antiphus* on their host plants *Aristolochia acuminata* and *Aristolochia cf. foveolata* through systematic monitoring of the Poring Butterfly Garden (Sabah, Malaysia). Here, we make the first observation of hypersensitive response (HR)‐like cell death and neoplasm formation in response to butterfly eggs deposited on *Aristolochia* leaves. This represents the first documented case of such egg‐killing traits in the highly diverse Magnoliids (> 10,000 spp.). HR‐like cell death under eggs showed a statistically significant negative effect on butterfly egg survival. Additionally, we provide a quantitative overview of the oviposition sites selected by Troidini, establishing that up to 37.6% of eggs are not oviposited on the host plant. Together, this suggests a fitness cost to butterflies and a possible behavioral avoidance mechanism. These findings on egg‐killing and oviposition behavior provide a significant contribution to the study of coevolutionary interactions between Troidini and *Aristolochia*. Furthermore, these observations provide valuable information for butterfly gardens and conservation projects, where egg‐killing by host plants may significantly impact butterfly survival. Overall, this research provides a unique egg‐based perspective on the ecological interactions between Troidini and their host plants.

## Graphical abstract

This study investigates the oviposition ecology of the iconic Troidini butterflies on their host plants of the genus Aristolochia in Sabah, where we made the first observation of HR‐like cell death and neoplasm formation in response to oviposition of Troidini, signifying the first such record in the entire Magnoliid clade (> 10,000 spp.). We provide a quantitative overview of Troidini egg survival and show the negative effects of HR‐like cell death on egg survival rates. Additionally, a large proportion of eggs was recorded as oviposited on structures other than leaves, suggesting a behavioral avoidance mechanism used by butterflies to escape egg‐killing by their host plants. HR‐like cell death reaction on *Aristolochia acuminata* leaf in reaction to an egg of *Pachliopta antiphus*.

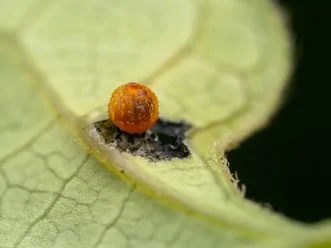

## 1 Introduction

Coevolution is one of the key driving mechanisms behind the formation of the Earth's biodiversity (Edger et al. 2015; Ehrlich and Raven 1964; Hembry et al. 2014; Speed et al. 2015). It occurs as a reciprocal process of adaptations and counteradaptations between interacting species. A classic example occurs between herbivorous insects and plants, where insect herbivory drives the diversification of plant anti‐herbivory chemicals, and these plant chemical defenses in turn drive the diversification of herbivorous insects (Becerra 2015; Beran and Petschenka 2024; Edger et al. 2015; Ehrlich and Raven 1964; Speed et al. 2015). A system that illustrates this is that of the Pieridae butterflies and Brassicales, where caterpillars evolved the ability to detoxify the plants' glucosinolates, with major innovations in this coevolutionary process being associated with gene and genome duplications in the butterflies and plants (Edger et al. 2015; Wheat et al. 2007). Examples of such interactions are widespread across the order Lepidoptera, including between Troidini caterpillars (Lepidoptera: Papilionidae) and their Aristolochiaceae host plants (Nishida et al. 1993; Nishida and Fukami 1989). Troidini larvae have adapted to detoxify toxic aristolochic acids (AAs) produced by host plant genera *Aristolochia* and *Thottea*, which allows them to feed on these plants (Nishida et al. 1993; Nishida and Fukami 1989). Additionally, several Troidini species can sequester these AAs in their larval stages and eggs, rendering them toxic to nonspecialized predators (Nishida et al. 1993; Nishida and Fukami 1989).

Plant anti‐herbivore defenses against insects have not only been found to affect actively feeding life‐stages but have also been shown to occur during the comparatively less studied egg stage (Griese et al. 2017, 2021; Hilker and Fatouros 2015). Different types of defense strategies against insect eggs have been recorded in different plants (Hilker and Fatouros 2015; Fatouros et al. 2016). An example of a defense mechanism is wound tissue growth, by which plants are able to crush eggs (Aluja et al. 2004; Desurmont and Weston 2011; Mazanec 1985). Additionally, eggs can induce leaf cell death resembling a hypersensitive response (HR) typically initiated by plant pathogens (Little et al. 2007; Shapiro and Devay 1987). In this case, the plant tissue surrounding the egg‐laying site becomes necrotic, often resulting in the desiccation of eggs (Fatouros et al. 2016). This reaction was first found in Brassicales and is hypothesized to be a counteradaptation of some brassicaceous species to the glucosinolate detoxifying abilities of Pieridae caterpillars (Hilker and Fatouros 2015). Neoplasm formation is another defense trait against eggs, where rapid growth of cells surrounding the egg can cause an egg to fall off the plant (Fatouros et al. 2016; Hilker and Fatouros 2015; Petzold‐Maxwell et al. 2011). At the time of writing this article, the expression of egg‐killing traits has been found in over thirty five plant species, spread over sixteen families (Bittner et al. 2017; Costa et al. 2024; de Oliveira Pinto et al. 2022; Fatouros et al. 2016; Li, Pei, et al. 2024; Lortzing et al. 2024; Da Song et al. 2024; Subramanyam et al. 2019; Wei et al. 2024). No records of leaf‐related egg‐killing traits have so far been documented for the entire Magnoliid plant clade, despite the presence of adaptations in Troidini and host plants that may have arisen through coevolutionary processes (Nishida et al. 1993; Nishida and Fukami 1989).

Here, we aim to address this knowledge gap by investigating possible egg‐killing traits of *Aristolochia* against the eggs of Troidini occurring in Sabah, Malaysian Borneo. Five genera of Troidini are found in Borneo: *Troides, Trogonoptera, Atrophaneura, Losaria* and *Pachliopta* (Chung et al. 2024). It is well known that these genera are oligophagous on host plant genera *Aristolochia* and *Thottea* (Böhm 2018a, 2018b, 2018c; Böhm et al. 2018; Matsuka 2001). Despite this, their exact host plant ranges are largely unknown due to the poorly studied taxonomy of the plant genera and the difficulty of studying host plant interactions in the field. *Aristolochia acuminata* Lam. is one of the well‐studied and well‐documented species serving as host plant for various Troidini (Böhm 2018b; Böhm et al. 2018; Haugum and Low 1979; Matsuka 2001; Straatman 1961). Moreover, this species is often used for captive breeding of these butterflies, particularly in conservation projects for threatened birdwings, the troidine genera *Troides* & *Ornithoptera* spp. (Peggie et al. 2025; Sutton et al. 2021).

We conducted a survey of Troidini butterfly oviposition on *Aristolochia* host plants in the Poring Butterfly Garden (Malaysian Borneo) and aim to address the following questions: (1) Does *Aristolochia* exhibit defense mechanisms against the eggs of Troidini? (2) To what extent do these defense mechanisms affect egg survival? and (3) Do Troidini exhibit oviposition counteradaptations to egg‐killing traits? In‐depth knowledge of the oviposition ecology can improve captive and semi‐captive breeding efforts and enable more efficient conservation efforts. Furthermore, knowledge on host plant–egg interactions and oviposition ecology will afford a more complete view of coevolutionary interactions between Troidini and their *Aristolochia* host plants.

## 2 Methodology

### 2.1 Study Site

We conducted this research in the Poring Butterfly Garden in Kinabalu National Park (Sabah, Malaysian Borneo), where a seminatural butterfly rearing is located on the edge of the rainforest (Figure 1, “Butterfly rearing”). The butterfly rearing is surrounded by a butterfly garden and planted with a high density of local butterfly nectar‐ and host plants. This provides a suitable study site for making life‐history observations of free‐living butterflies which are attracted from the adjacent rainforest (Figure 1, “Butterfly garden”). The host plants of local Troidini butterflies, *Aristolochia acuminata* Lam. and *Aristolochia cf. foveolata* Merr., are cultivated in the gardens. Despite *A. foveolata* being mentioned frequently by botanical and lepidopterological literature on Borneo (Chung et al. 2024; Haugum and Low 1979; Hou 1984; Matsuka 2001; Sutton et al. 2021), there is increasing support that *A. foveolata* may in fact be absent from Borneo and may belong to one of several similar‐looking species in the “*papillifolia* group” (Frank et al. 2026). We refer readers to Frank et al. (2026) for a detailed taxonomic perspective but continue to refer to the studied taxon as *A. cf. foveolata* in this manuscript following the identification key in (Hou 1984). The host plants grew on trellises that were approximately between 160 and 185 cm tall. A roughly estimated one third of the butterfly garden was occupied with *Aristolochia* plants, in a patch where *A. acuminata* contributed 75% and *A. cf. foveolata* 25% of vegetation cover. The most commonly occurring Troidini species are *Troides helena* (Linnaeus, 1758), *Troides amphrysus* (Cramer, 1782), *Trogonoptera brookiana* (Wallace, 1855) and *Pachliopta antiphus* (Fabricius, 1793).

**FIGURE 1:**
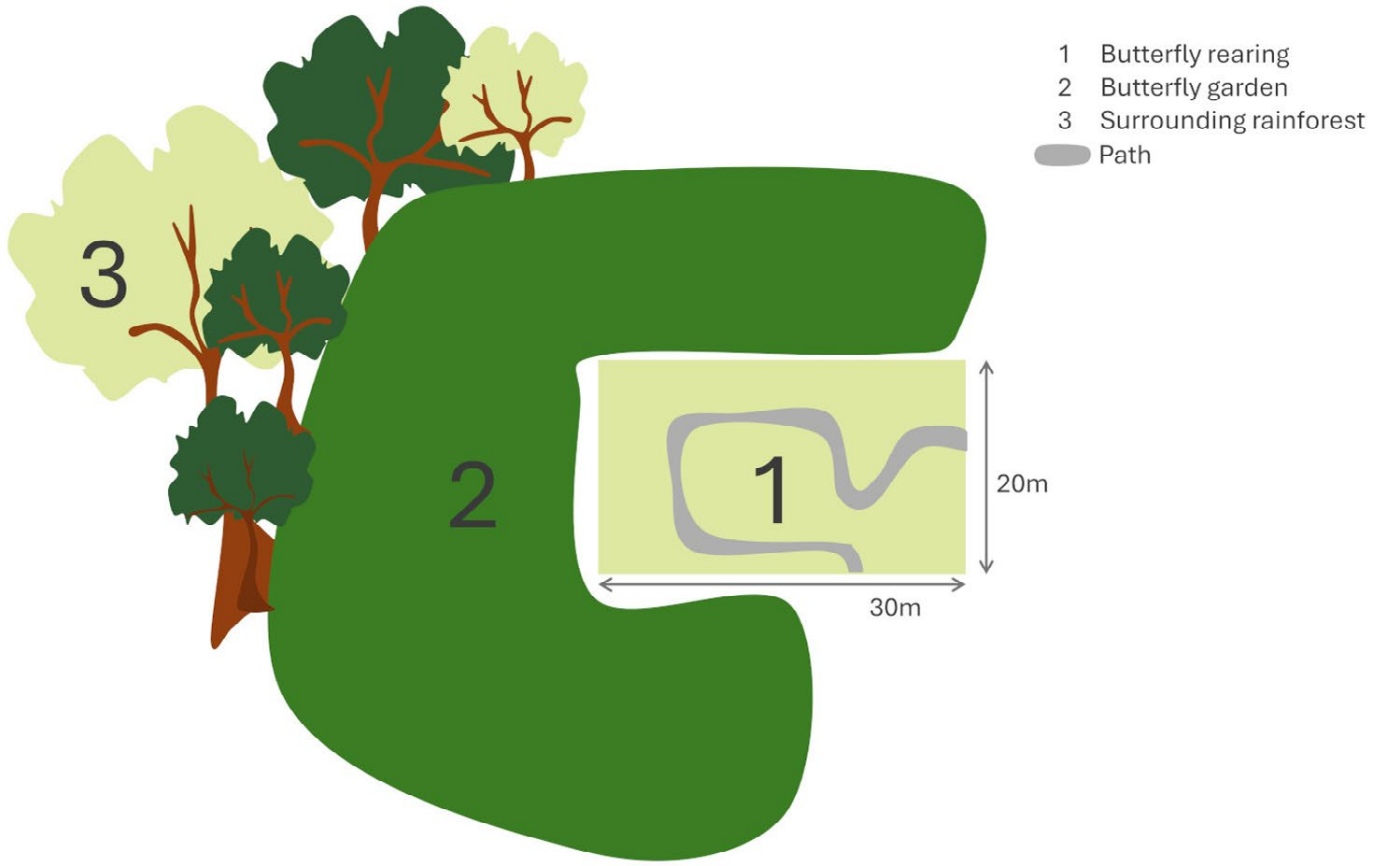
Poring study site map. The butterfly rearing and the butterfly garden are expressed in relative sizes. The whole area is flanked by primary rainforest.

### 2.2 Survey of Butterfly Oviposition Location and Resulting Plant Responses

From the 4th of March to the 22nd of April 2024, the butterfly garden was monitored for eggs of the Troidini clade. Initially, oviposition sites and plant responses were surveyed opportunistically. Eggs were collected to monitor egg survival rates, in total allowing 86 eggs to be surveyed. After making the initial observations of HR‐like cell death and neoplasm formation, a 10‐day survey of direct egg‐killing traits was performed from the 13th to the 22nd of April. During this daily survey, a directed search was carried out for newly deposited eggs. Both the adaxial and abaxial leaf sides were checked for newly deposited eggs, as well as the stem and support material (trellises and support sticks) of all *A. acuminata* and *A. cf. foveolata* individuals present in the garden. When an egg was encountered, the plant on which it was found was marked with a code, the location of oviposition was noted, and the egg was collected. If multiple eggs were present on the same leaf, all eggs were collected at the same time. Egg placement on and near host plants *A. acuminata* and *A. cf. foveolata* was divided into seven categories: dead stem, stem, adaxial and abaxial side of the leaf, other plant and “other” for oviposition on non‐plant material. When HR‐like cell death was expressed underneath an egg, a picture of the adaxial and abaxial side of the leaf was taken and the intensity of the response was later scored based on the picture. When HR‐like cell death was present, eggs were collected together with leaves. After collection, the eggs were kept in plastic containers of approximately 20 × 10 × 5 cm. Containers were kept at room temperature (approximately 23°C) and checked daily for the emergence of caterpillars. Each egg was identified to species level based on morphological characteristics such as size and color.

### 2.3 Scoring and Statistics

Pictures of eggs inducing HR‐like cell death were scored on a scale from zero to three in a modified version of the system used by Griese et al. (2017). Scores of zero were given to leaves that displayed no visible response (Figure 2). The score of one was allocated to leaves where HR‐like cell death was only present in a small circle under the egg and not on the opposite side of the leaf. Leaves where HR‐like cell death was present under the eggs and where it was visible on both sides of the leaf were classified as score two. Leaves on which the HR‐like cell death had spread from under the eggs and could be seen clearly on both sides of the leaf were scored as three. Additionally, the presence of cell growth/neoplasm formation beneath the eggs was separately scored as one for presence and zero for absence.

**FIGURE 2:**
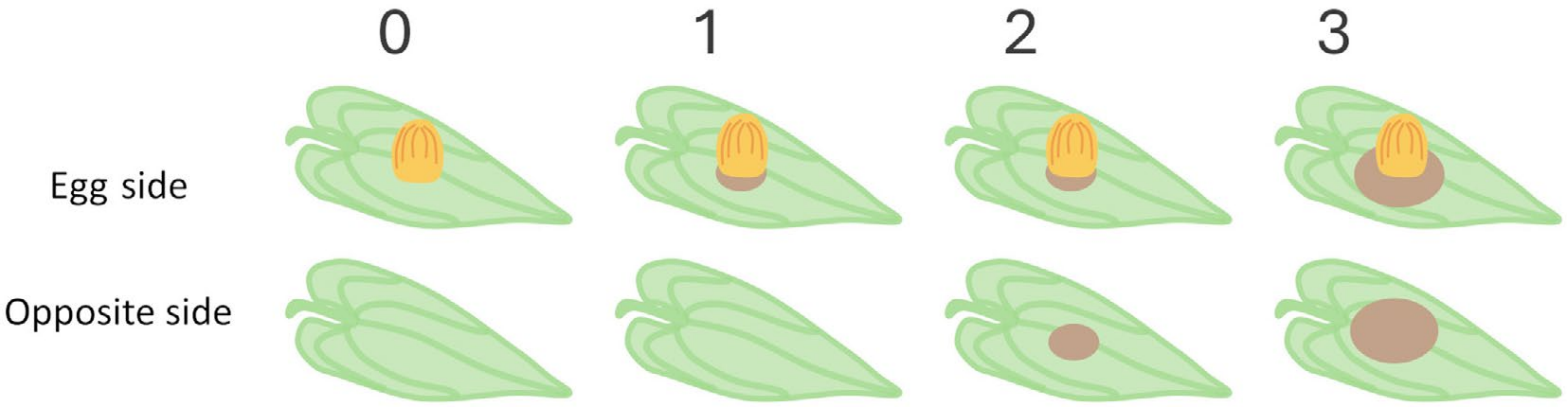
Scale for scoring cell death. 0. No cell death. 1. Cell death is only present in a circle under the egg and not on the opposite side of the leaf. 2. Cell death under the eggs and visible on both sides of the leaf. 3. Cell death has spread from under the eggs and can be seen clearly on both sides of the leaf.

After this, the effect of HR‐like cell death formation on egg survival was tested by using a binomial generalized linear model (Bates et al. 2015). Neoplasm formation was not included in this analysis due to the low sample size. Butterfly species and HR‐like cell death were tested as additive predictors. This was done using the *nnet* package version 7.3.20 (Venables and Ripley 2002) in R version 4.5.0 (R Core Team 2025). All observations of eggs made between the 4th of March and the 22nd of April 2024 were included for the egg survival rate analysis. *Troides amphrysus* could not be included in this model because of the limited sample size.

## 3 Results

### 3.1 Egg Placement

We were able to record egg placement for three species of butterflies, *P. antiphus*, *T. amphrysus*, and *T. helena* , in the Poring butterfly garden on and near host plants *A. acuminata* and *A. cf. foveolata* (Figures 3, 4, 5). The survey showed that eggs were not only laid on the adaxial and abaxial sides of leaves on the plants, but also on stems and petioles (Figures 3 and 4). Oviposition location varied between plant and butterfly species. Where *T. amphrysus* only oviposited on the abaxial side of leaves of both host plants, *P. antiphus* oviposited on the petiole of both host plants, while the other butterfly species did not, and *T. helena* was the only species to oviposit on a stem (Figure 4). Oviposition patterns also differed for host plant species of both *P. antiphus* and *T. helena* (Figure 4).

Next to this, we noted that up to 37.6% of eggs were not laid on the *Aristolochia* host plants, but on surrounding structures (other) and, in the case of *T. helena* , even on surrounding non‐*Aristolochia* plants (Figures 3H and 5).

**FIGURE 3:**
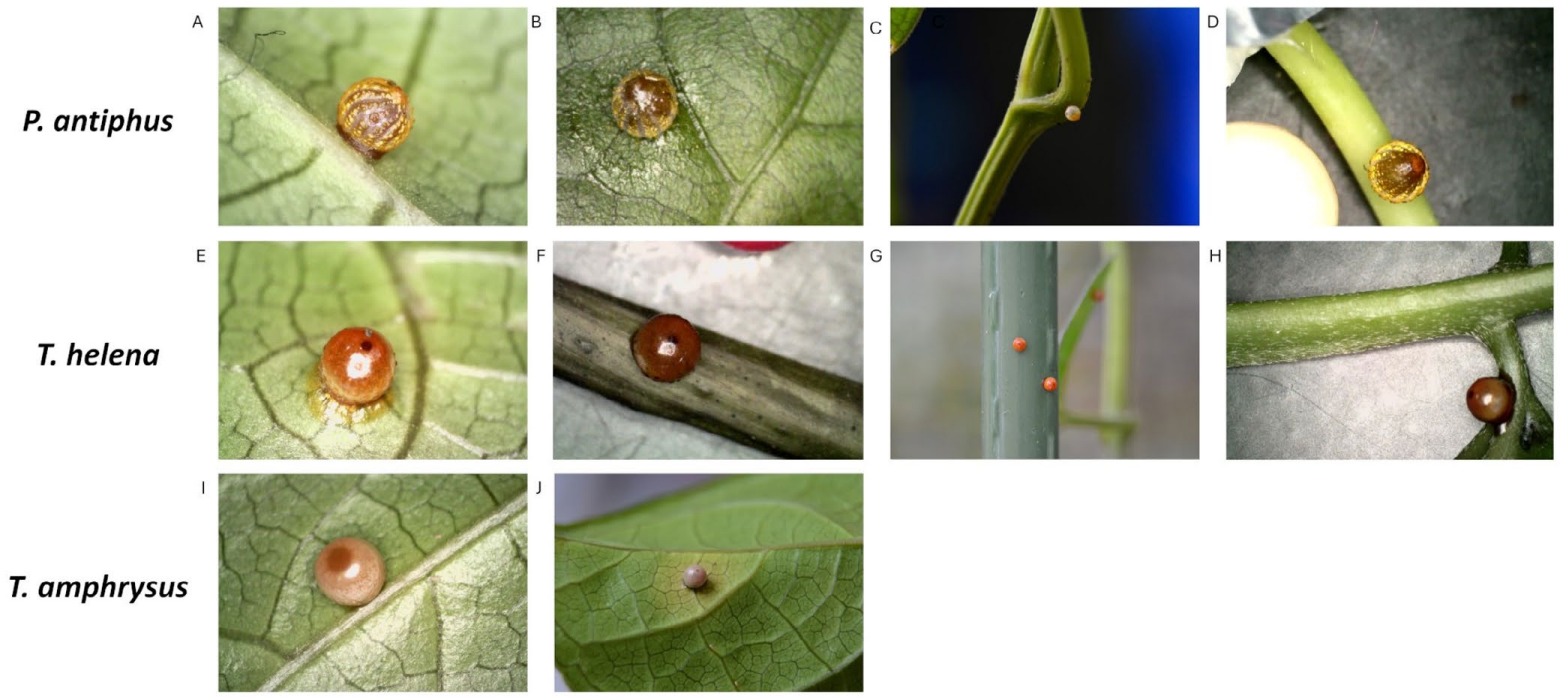
Placement of butterfly eggs on the host plants *A. acuminata* and *A. cf. foveolata*. Rows represent different butterfly species. (A) Egg of *P. antiphus* deposited on the abaxial side of a leaf of *A. acuminata* . (B) Egg of *P. antiphus* deposited on the adaxial side of a leaf of *A. acuminata* . (C) Egg of *P. antiphus* deposited on the petiole of *A. acuminata* . (D) Egg of *P. antiphus* deposited on the petiole of *A. cf. foveolata*. (E) Egg of *T. helena* deposited on the abaxial side of an *A. acuminata* leaf. (F) Egg of *T. helena* deposited on the stem of *A. acuminata* . (G) Two *T. helena* eggs oviposited on a plastic plant support stick. (H) Egg of *T. helena* oviposited on the petiole of a nonhost plant. (I) Egg of *T. amphrysus* deposited on the abaxial side of a leaf of *A. acuminata* . (J) Egg of *T. amphrysus* deposited on the abaxial side of a leaf of *A. cf. foveolata*.

**FIGURE 4:**
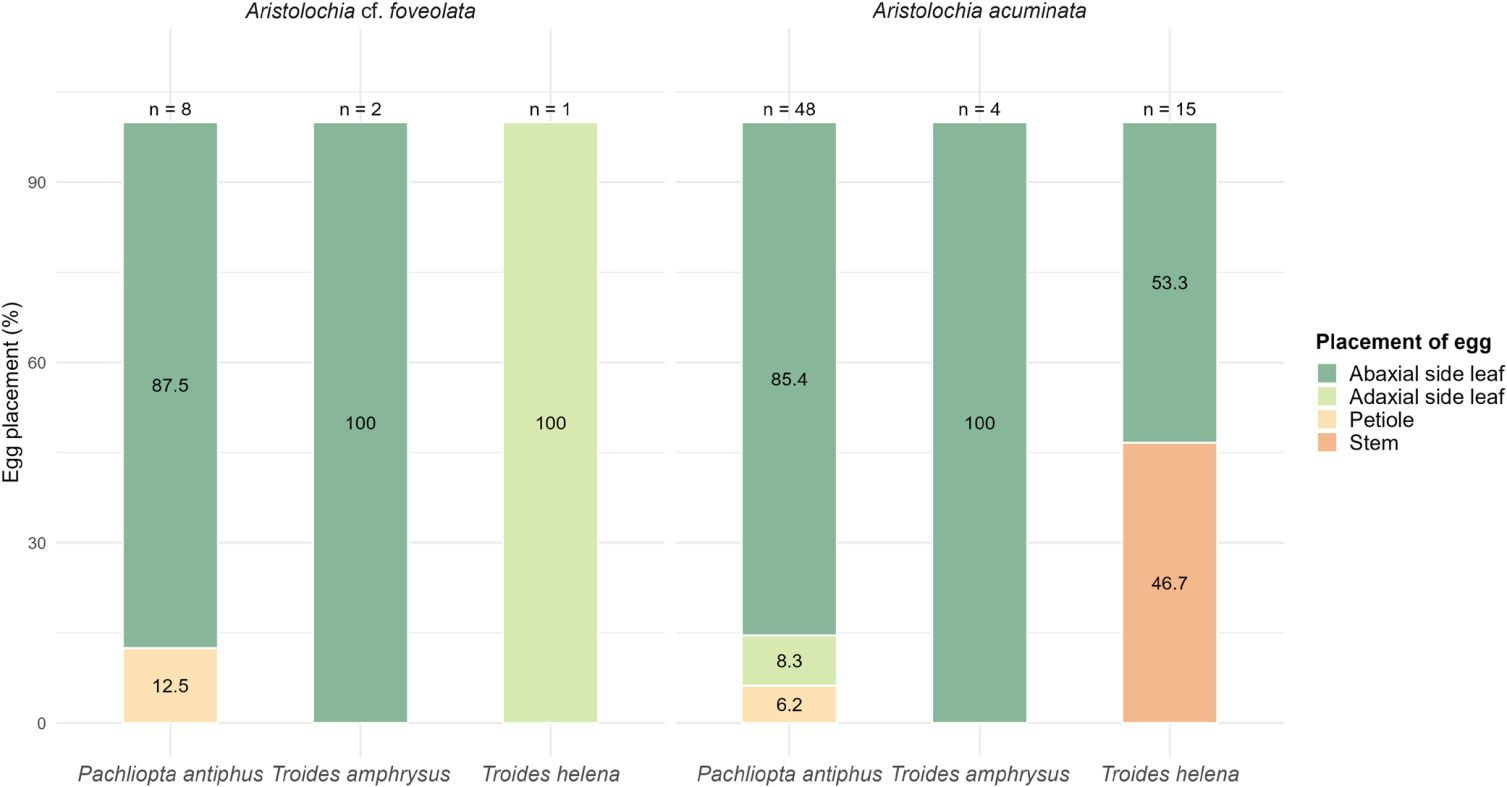
Egg placement percentages on *Aristolochia* host plants per butterfly species. The sample size (*n*) per category is indicated.

**FIGURE 5:**
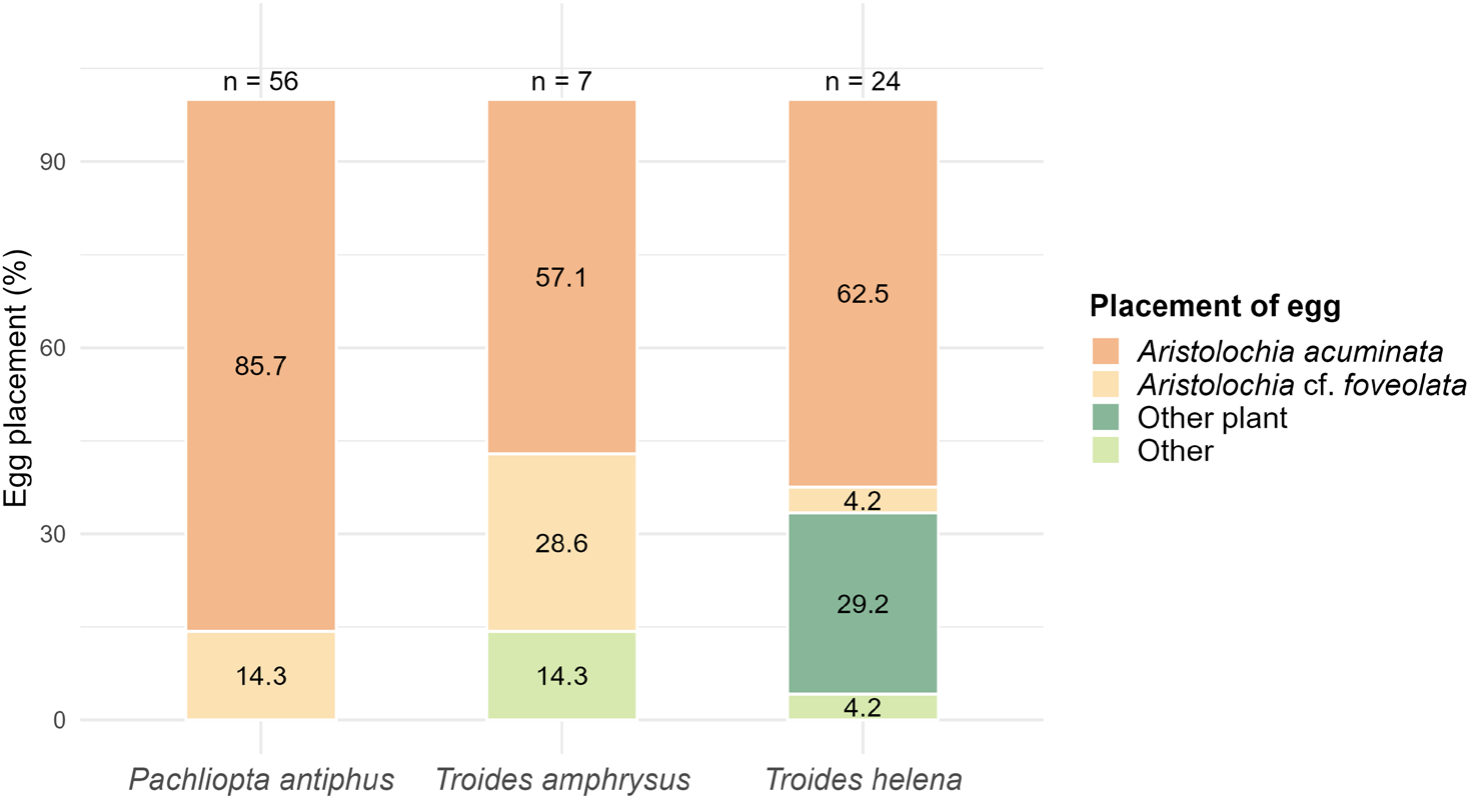
Egg placement percentages per butterfly species per location on and near the host plants *A. acuminata* and *A. cf. foveolata*. The “Other” placement category means another structure that is not a plant and “Other plant” is another plant species that is not a member of the Aristolochiaceae. The sample size (*n*) per category is indicated.

### 3.2 HR‐Like Cell Death and Neoplasm Formation

We recorded 23 observations of HR‐like cell death and 8 observations of neoplasm formation over the total duration of 1 month of research. For the cell death responses, the tissue surrounding the eggs initially turns yellow, progresses to brown, and eventually turns black upon dying. Neoplasm formation is when tissue growth occurs under the eggs, raising the egg up in relation to the leaf. Both HR‐like cell death and neoplasm formation were only detected on *A. acuminata* and on the abaxial side of the leaves (Figure 6).

**FIGURE 6:**
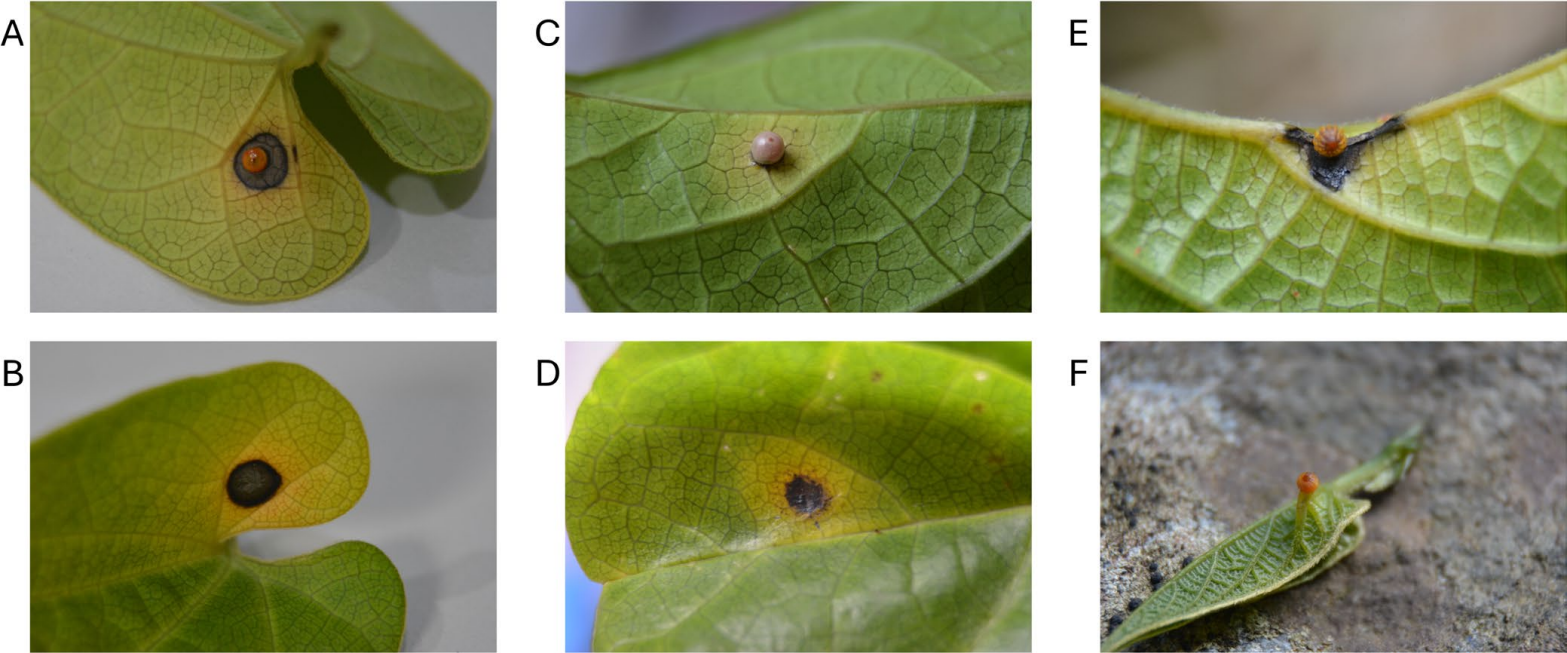
Pictures of HR‐like reactions in *A. acuminata* . (A) *T. helena* egg on the abaxial of a leaf surrounded by scale 3 cell death reaction. (B) Adaxial side of the leaf in picture A. (C) *T. amphrysus* egg on the abaxial side of a leaf surrounded by score 3 cell death. (D) Adaxial side of the leaf in picture C. (E) *P. antiphus* egg on the abaxial side of a leaf surrounded by score 3 cell death reaction. (F) *P. antiphus* egg on the abaxial side of a leaf with the presence of neoplasm formation and score one cell death.

Out of all 23 responses, 22 scored a severity of three for cell death, where it is visible from both sides of the leaf and cell death spread from under the eggs. The only response that scored a one for cell death was recorded on a developing leaf (Figure 6F). Neoplasm formation was also recorded on this leaf. Unfortunately, the leaf dried out immediately after collection, preventing further monitoring of the egg‐induced response. Not all eggs deposited on a plant that expressed cell death induced a cell death response. On five plants that previously showed HR‐like cell death induced by *P. antiphus* or *T. helena* eggs, we also recorded eggs that did not trigger any HR‐like cell death in the plant.

**FIGURE 7:**
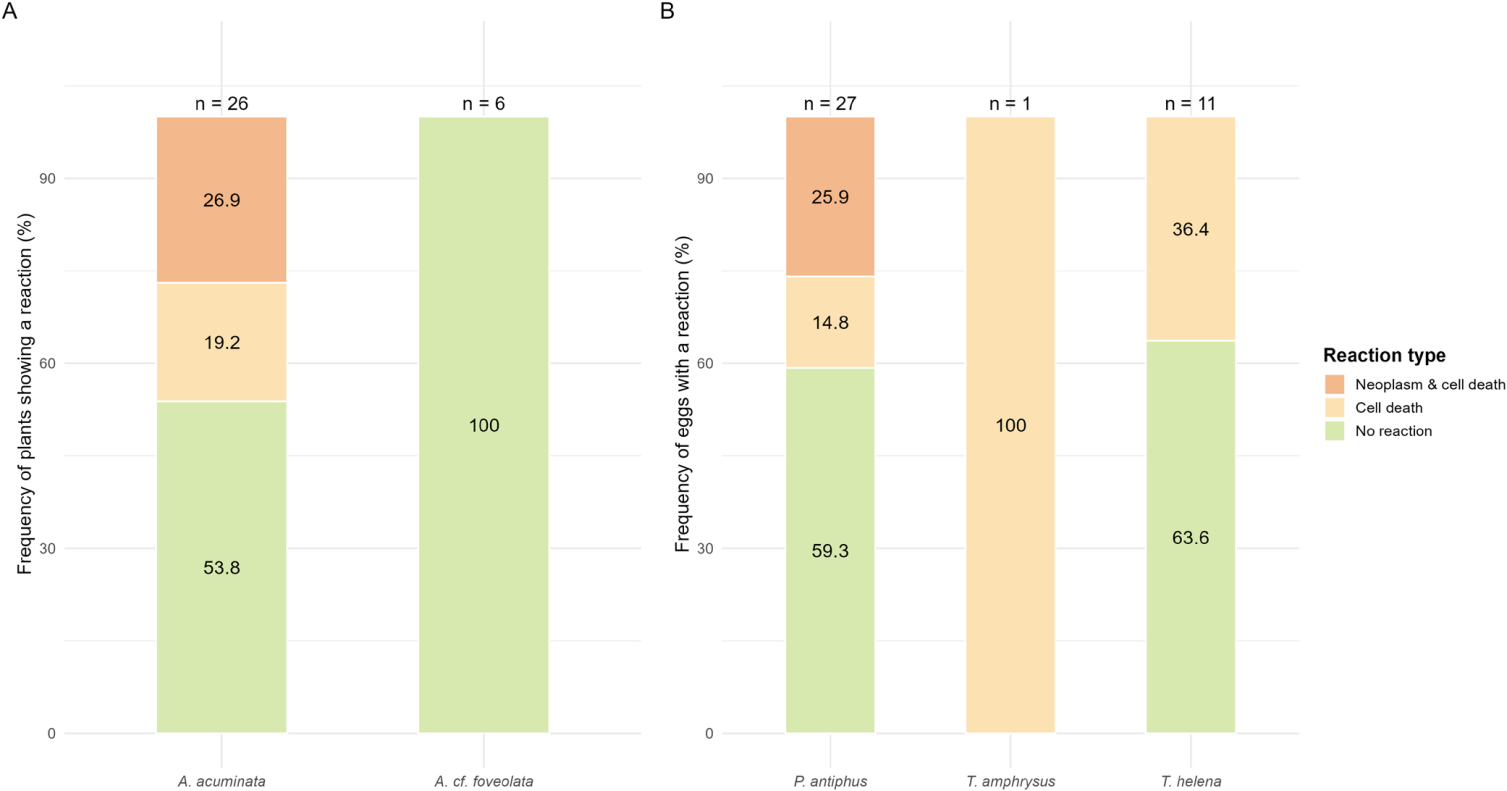
Bar plot of percentage of plants showing reactions and butterfly eggs with the occurrence of HR‐like cell death and neoplasm formation. (A) The percentage of eggs that showed a reaction per plant. (B) Percentage of eggs per butterfly species with a reaction on *A. acuminata* . The sample size (*n*) per category is indicated.

During a 10‐day survey focusing on the placement of eggs, we labeled 26 *A. acuminata* and six plants of *A. cf. foveolata*. In total, 12 plants of *A. acuminata* developed an HR‐like cell death (Figure 7A). Neoplasm formation, if encountered, was always found in combination with cell death (Figure 7). We recorded a total of 39 eggs belonging to *P. antiphus*, *T. amphrysus*, and *T. helena* on *A. acuminata* (Figure 7B). Neoplasm formation was only found for eggs of *P. antiphus* (*n* = 7).

We found a significant effect on egg survival rates by butterfly species and cell death (Figure 8). *P. antiphus* had a significantly higher probability to emerge as a caterpillar than the reference species, *T. helena* (Binomial model, log odds = 1.7656, *p* = 0.0334, Figure 8). For the eggs where HR‐like cell death was present, the odds of a caterpillar emerging were lower than the reference without cell death formation (Binomial model, log odds = −1.3168, *p* = 0.0458, Figure 8).

**FIGURE 8:**
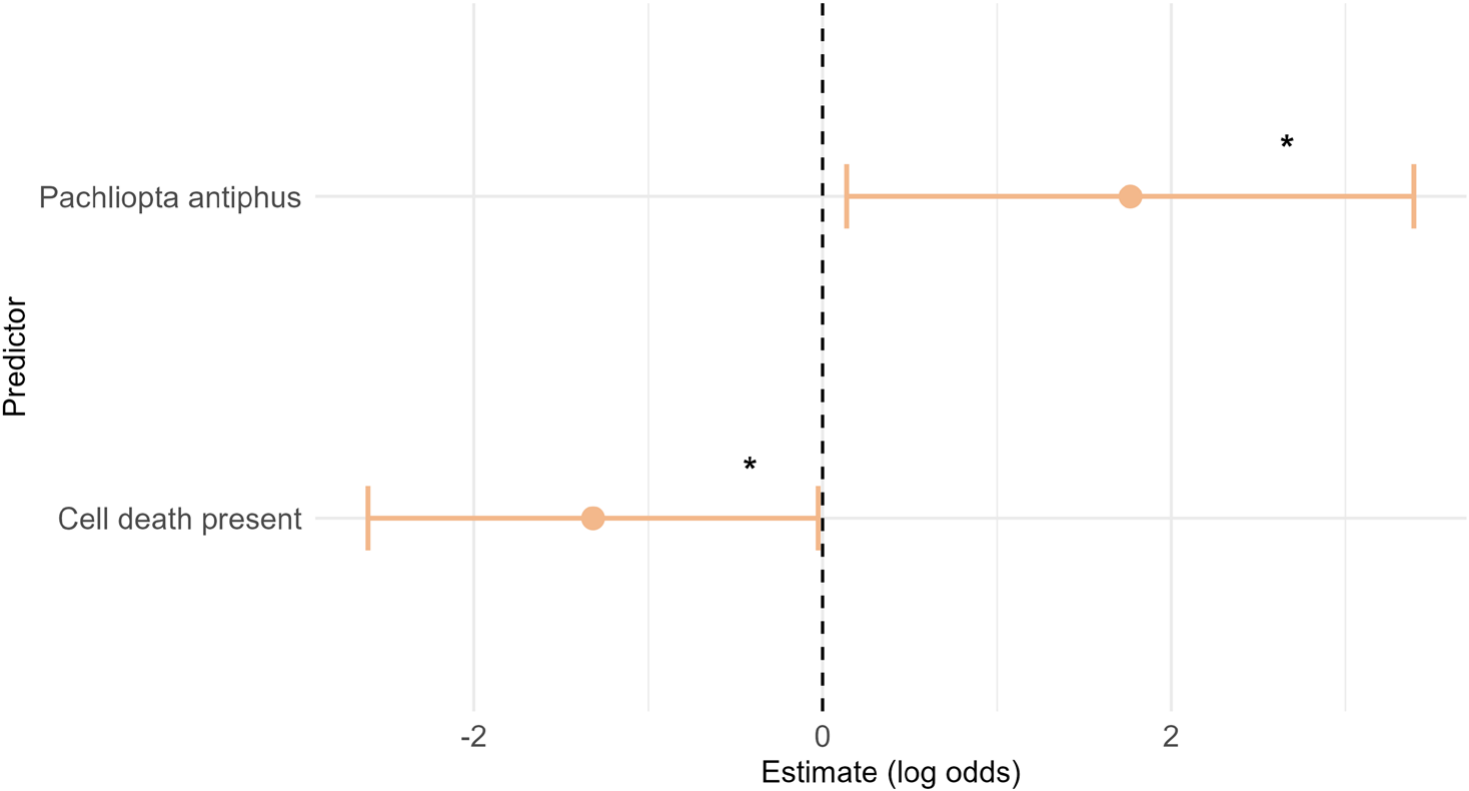
Visualization of the logistic model of the influence of butterfly species and cell death on caterpillar egg survival rates. Points represent model estimates in log odds, horizontal bars include the 95% confidence intervals. Asterisks indicate significant effects (*p* < 0.05). The dashed vertical line at zero represents no effect. The Intercept was not shown (estimate = −1.49, SE = 0.78, *p* = 0.057). *Pachliopta antiphus* had a positive effect on egg survival (estimate = 1.77, SE = 0.83, *p* = 0.033), whereas the presence of cell death had a negative effect (estimate = −1.32, SE = 0.66, *p* = 0.046).

Egg survival rates were split per butterfly species and the absence and presence of cell death; rates ranged between 22% and 56% (Figure 9). No eggs of the species *T. helena* hatched when cell death was present (Figure 9). Out of a total of seven eggs with neoplasm reactions recorded during this study, two caterpillars emerged. Neoplasm formation was not included in the figure and model due to low sample size.

**FIGURE 9:**
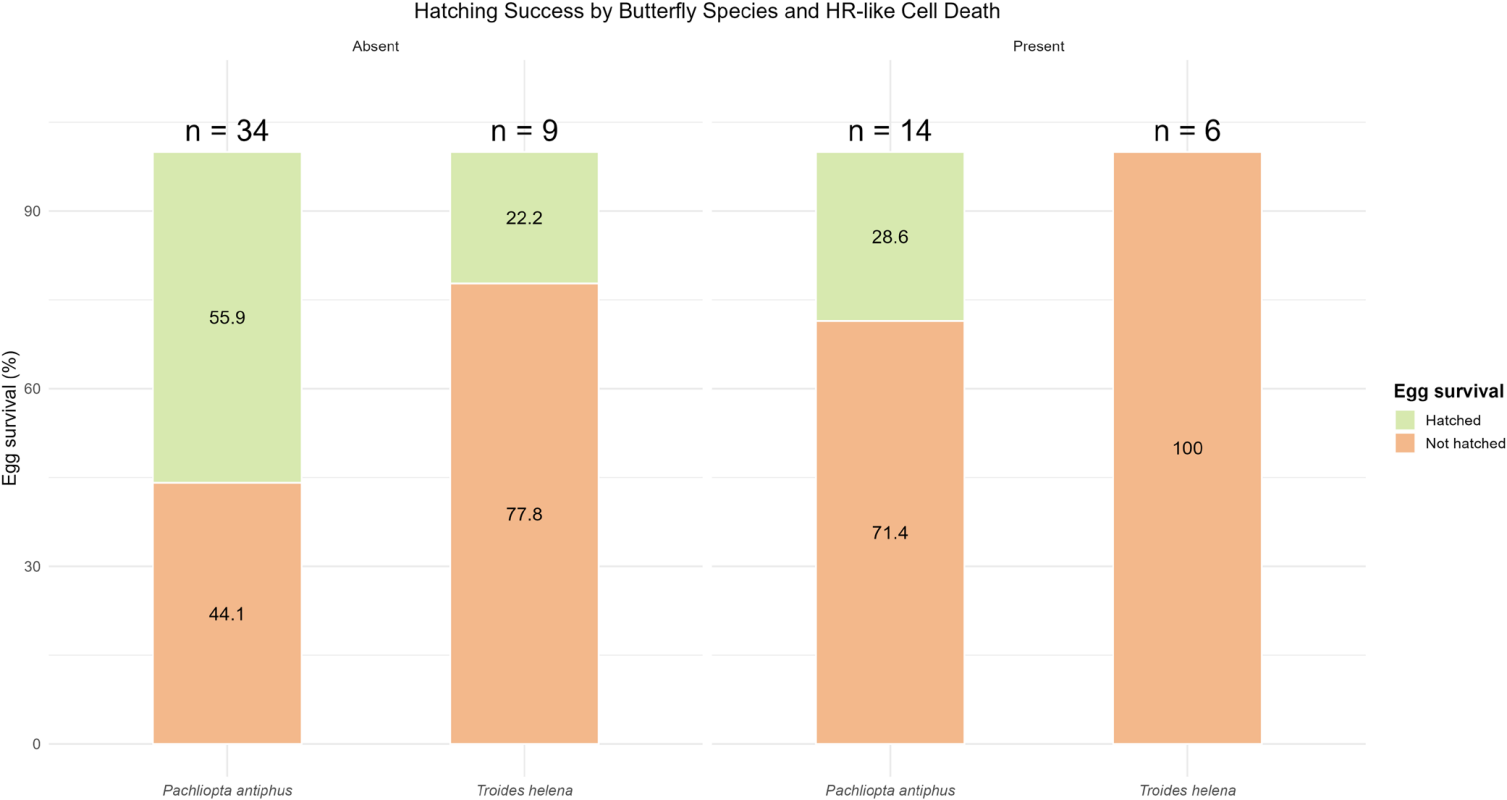
Bar plot of percentage of egg survival for eggs with the presence (left) or absence (right) of HR‐like cell death. The sample size (*n*) per category is indicated.

## 4 Discussion

We surveyed oviposition of Troidini butterflies in the Poring Butterfly Garden where we recorded egg placement and novel plant responses to butterfly eggs. Our observations provide the first record of HR‐like cell death and neoplasm formation in *Aristolochia acuminata*, and this reaction was induced by eggs of three species of Troidini: *T. helena* , *T. amphrysus*, and *P. antiphus*. Next to this, we statistically confirm a negative effect of the HR‐like cell death on egg survival, highlighting that this reaction may be an effect of coevolutionary processes between the egg stage of Troidini and *Aristolochia plants*.

### 4.1 First Record of HR‐Like Cell Death and Neoplasm Formation in the Magnoliids

As of 2016, 22 plant species were shown to express egg‐induced direct defense traits (Fatouros et al. 2016), with an additional 9 species recorded since then (Bittner et al. 2017; Costa et al. 2024; Li, Pei, et al. 2024; Lortzing et al. 2024; Da Song et al. 2024; Subramanyam et al. 2019; Wei et al. 2024). To our knowledge, our observations provide the first record of leaf‐related defense strategies against the egg stage of insects in the Magnoliids (> 10,000 species). Until now, wound tissue growth in the avocado ( *Persea americana* Mill., Lauraceae) in response to flies of the genus *Anastrepha* in Mexico was the only known observation of plant defense against insect eggs within the Magnoliids (Aluja et al. 2004). In *P. americana* , wound tissue growth was found on the fruit, which was mechanically damaged by the hole‐boring activity of ovipositing *Anastrepha* flies. We observed egg‐induced response only on leaves, which were not mechanically damaged by butterfly oviposition. The effects of the responses also differed; wound tissue growth mechanically crushes eggs, while HR‐like cell death likely causes egg desiccation and/or eggs falling off the leaf, and neoplasm formation is thought to push eggs off the plant (Aluja et al. 2004; Fatouros et al. 2016). These differences illustrate that the formation of HR‐like cell death and neoplasm are distinct defense mechanisms, making our discovery the first documented case of these responses in the Magnoliids.

The complexity of the mechanisms of direct defense traits is not only shown by interspecies relationships but is also underscored by intraspecific variation. Not all *A. acuminata* plants consistently expressed cell death, and this phenotypic variation may be due to underlying genetic variation as all plants were grown under common conditions. The provenance of the studied population of *Aristolochia* is unknown, as plants have been transplanted from a small number of “donor” plants growing in the wild, obscuring insights into intraspecies variation of the traits investigated. Comparably, different *Mutarda nigra* Bernh. populations were found to show phenotypic variation in HR‐like cell death (Bassetti et al. 2024). Next to intraspecific variation, variation in response severity was detected on the same plant, with some eggs from the same butterfly species eliciting a response while others did not. This could possibly be explained by differences in egg‐casing chemistry among ovipositing butterflies, which may trigger reactions and influence their severity or presence. Seeing as the AA content per butterfly has been shown to differ according to dietary intake, individual butterflies may also differ in their concentrations of other plant‐derived chemicals (Dimarco and Fordyce 2017). Chemistry of leaves also changes as they age and grow (Jeong et al. 2023), introducing another possible source of variation in responses within an individual plant. Next to this, consistently non‐reacting plants may potentially be explained by a time bias in our study. Plants were surveyed daily, so responses might not have developed completely at the time of making observations. However, the only egg found in the initial stage of cell death (score = one) was found on a very young leaf, suggesting that the responses likely start quickly but take some time to reach the full extent. However, the plants were surveyed once daily, so at least in some cases, the reaction is able to reach full severity (score = 3) within 24 h.

Our research is constrained by its observational design, single‐location, seminatural, and butterfly garden setting, which may influence butterfly oviposition behavior. The high *Aristolochia* densities at this location facilitated the observation of oviposition behavior but may not accurately reflect oviposition patterns in nature. Quantitatively studying responses to butterfly oviposition on multiple individuals of *Aristolochia* plants in their natural environment would allow for understanding this trait in the wild. Furthermore, combining such studies with genomic studies of plants from different populations would enable the study of intra‐specific phenotypic and genetic variation. Common garden experiments with *Aristolochia* from a known genetic background and experimentally exposing these to ovipositing butterflies would allow for the study of the variation in response between and within plants.

### 4.2 Possible Coevolutionary Interactions Between *Aristolochia* and Troidini Eggs

*Aristolochia* also has defense traits that act on the caterpillars of Troidini, including trichomes influencing feeding behavior of caterpillars and diterpenoid compounds causing differential gene expression in gut and fat body tissues (Fordyce and Agrawal 2001; Silva‐Brandão et al. 2025). Our findings broaden the defense traits in this context to the egg stage, with HR‐like cell death being associated with lower egg survival for the species *T. helena* and *P. antiphus* (Figure 8). Overall, *P. antiphus* had higher odds of egg survival compared to *T. helena* (Figure 8). The eggs of *P. antiphus* had an egg survival rate of 56% on non‐responding leaves, compared to 29% where HR‐like cell death was present (Figure 9). Eggs of *T. helena* had a survival rate of 22% on non‐responding leaves, whilst no eggs hatched under the presence of HR‐like cell death (Figure 9). In the *Pieris* Brassicaceae study system it is well‐established that HR‐like cell death negatively influences egg survival through the desiccation of eggs (Fatouros et al. 2012, 2014; Griese et al. 2021; Peters et al. 2024). Caterpillars of Troidini are known to consume large amounts of leaf tissue during development (Alam et al. 2014; Barua and Slowik 2007), even defoliating entire plants (Straatman 1971), and reduced survival in the egg stage most likely confers a significant benefit for the plants.

Larger sample sizes and longer monitoring of eggs on the plant is necessary to fully understand the fitness effects of this interaction on both butterfly and host plant. It would also be interesting to characterize the chemical composition of these lesions to gain more understanding of chemical processes involved. Additionally, a comprehensive survey of egg‐killing traits across *Aristolochia* species and co‐occurring Troidini species, combined with phylogenetic information for both groups, could elucidate a broader coevolutionary pattern between host plants and herbivore eggs.

### 4.3 Egg Placement: Behavioral Adaptations as a Result of Coevolutionary Interactions?

During our studies, we observed a relatively high proportion of eggs not laid on host plant leaves. On *A. acuminata* , 6.2% of the detected *P. antiphus* eggs were oviposited on petioles, while *T. helena* deposited 46.7% of eggs on stems (Figures 3 and 4). Additionally, we observed that eggs were also oviposited on structures other than host plants: *T. amphrysus* oviposited 14.3% of detected eggs on nearby non‐plant structures, and 4.3% of eggs contributed to this category for *T. helena* . Moreover, *T. helena* oviposited 29.2% of its observed eggs on plants not belonging to the genus *Aristolochia* (Figures 3H and 5). The few sources documenting Troidini oviposition solely mention the abaxial and adaxial sides of leaves or stems of host plants as oviposition locations (Alam et al. 2014; Barua and Slowik 2007; Böhm et al. 2018; Haribal 1992; Straatman 1961; Straatman and Schmid 1975). Straatman (1961) mentions that eggs of *T. brookiana* ovipositing on *Aristolochia* sp. can be found up to 20 cm from the host plant on dead twigs or other plants. A recent study on *T. amphrysus* in Java identified *A. zollinggeriana* Miq. as a new host plant, noting that most eggs were deposited on other plants near this host plant (Shahroni et al. 2025). Not only do we also find a diverse array of oviposition sites used by *T. helena* , *T. amphrysus* and *P. antiphus*, but our results also provide a rare quantitative perspective. Taken together, this suggests that oviposition on nonhost plant sites may be relatively common across the Troidini. Based on our observations on egg survival in response to defense traits in leaves, we cautiously attribute this behavior to be a potential “avoidance mechanism” used by the butterflies to escape HR‐cell death response and neoplasm formation in *A. acuminata*. Several species within the Pieridae seem to exhibit a similar oviposition preference when exposed to HR‐like cell death expressing Brassicaceae (Griese et al. 2021), suggesting counteradaptation by the butterflies (Peters et al. 2024).

Other drivers of the observed oviposition patterns may be the selection of more concealed oviposition locations by butterflies to avoid predation (Fatouros et al. 2020). Differing microclimate conditions around the hostplant may also determine the selection of oviposition sites (Eilers et al. 2013). Additionally, Satyrinae species with superabundant hosts often do not deposit their eggs on leaves their larvae will feed on as they can easily be found by the neonate caterpillars (Wiklund 1984). An alternative explanation is that females may oviposit on several nonhost species before they find their preferred host if these have similarly shaped leaves (Wiklund 1984). *Aristolochia* is not superabundant in the wild, and has visually distinct leaves, but the density of plants in the butterfly garden could influence oviposition behavior. Furthermore, another Troidini butterfly, Battus polydamas (Linnaeus, 1758), has been shown to locate its host through olfactory and tactile cues, suggesting that visual identification may be less important in Troidini oviposition (Pinto et al. 2009). Despite not being able to experimentally rule out other mechanisms, our observations serve as an indication of a behavioral counteradaptation to egg‐killing host plant traits. Understanding of the extent of fitness effects on Troidini butterflies and their host plant must be established to fully understand the complexity of this interactive relationship.

### 4.4 New Insights Into Egg Survival Rates of Troidini Butterflies

In this survey, we were able to record the egg survival rates of *T. helena* (22%) and *P. antiphus* (56%, Figure 9), providing one of the few overviews of egg survival in Troidini to date. To accurately determine the extent of the effects of HR‐like cell death and neoplasm formation on egg survival, baseline knowledge on egg survival rates of Troidini in the wild would be advantageous. However, literature on the egg survival rates of Troidini is scarce since eggs are difficult to locate and monitor in the wild. The only available study on Troidini egg survival rates was performed on *Troides aeacus* (Felder, 1860), which was reported to peak at 40% survival for an egg clutch and went as low as 5.7%, showing a similar range to our rates for different butterfly species (22%–56%) (Hsieh et al. 2010). *T. aeacus* lays eggs in clusters, while the species in our study laid solitary eggs. This difference in oviposition strategy may influence egg survival, thus not making these rates directly comparable, but rather providing a general reference. Another example from within the Papilionidae but outside the Troidini is a study on *Papilio godeffroyi* (Semper, 1866), reporting an egg survival rate of 14.7%, slightly below the mean of our own measurements (Banko et al. 2024). Next to improving our knowledge on butterfly egg‐plant relationships, information on such is also valuable for breeding programs of threatened species within the Papilionidae.

### 4.5 Informing Conservation Efforts

Because of international trade, drastic habitat loss and habitat quality decline, the IUCN has classified several Troidini species as vulnerable (Benedick et al. 2006; Böhm et al. 2018; Gaveau et al. 2014; IUCN 2025). In response, conservation efforts have been implemented for several species (Swallowtail and Birdwing Butterfly Trust 2026). In Sabah, these efforts have been focused on the endemic Kinabalu birdwing (*Troides andromache*), recently proclaimed as Sabah's state butterfly (mysabah.com 2023; Sabah Biodiversity Centre 2023; Sutton et al. 2021; Swallowtail and Birdwing Butterfly Trust 2026). Conservation measures implemented by the Kinabalu Birdwing Butterfly project include the propagation of host plants to bolster naturally occurring populations, planting of nectar gardens and the development of education packages and ecotourism with *T. andromache* as flagship species (Sutton et al. 2021; Swallowtail and Birdwing Butterfly Trust 2026). To properly inform such conservation initiatives it is crucial to gather more information on their ecological interactions (Benedick et al. 2006; Böhm et al. 2018; Bonebrake et al. 2010; Gaveau et al. 2014). Knowledge on egg‐killing traits is key in breeding projects and can be used to ensure that only non‐expressing host plants are planted in butterfly gardens and to support wild butterfly populations. Additionally, our research provides information on egg survival rates, which can be used as a reference point for alerting to possible problems related to egg survival rates.

Complementary to aiding local conservation efforts in Sabah, the knowledge gained from our survey can be transferred to conservation projects for other Troidini. For example, the conservation project of the near‐threatened Wallace's birdwing, *Ornithoptera croesus* (Wallace, 1859), endemic to North Maluku (Indonesia) (Böhm 2018b). Seminatural breeding efforts for this species rely on *A. acuminata* as a host plant, instead of the more difficult‐to‐cultivate wild host plants of the species, which belong to the *Pararistolochia* group of *Aristolochia* (Peggie et al. 2025). Here, potential egg‐killing by *A. acuminata* could impact egg survival and reduce the effectiveness of captive breeding. Our insights may also aid in the conservation of the enigmatic Queen Alexandra's Birdwing (*Ornithoptera alexandrae* (Rothschild, 1907), classified as threatened by the IUCN). Additionally, it has been reported that in experimental settings these butterflies mature well, and even faster on the more easily available *A. acuminata* (Straatman 1971). However, survival of eggs has not been quantified, and may be lower on *A. acuminata* opposed to their naturally occurring host plants, depending on the presence of if egg‐killing traits. It is unknown if HR‐like expressing plants are used in the breeding programs of these threatened butterfly species, but this warrants investigation as it may significantly reduce conservation breeding success. Understanding the interactions of these butterflies and their hosts, and in particular the oviposition behavior and egg‐killing traits of their hosts, is imperative for successful conservation strategies.

## 5 Conclusion

In this study, we observed the presence of defense mechanisms in *Aristolochia acuminata* in response to eggs of several Troidine butterfly species. To our knowledge, this observation marks the first record of such mechanisms on leaves within the Magnoliids (> 10,000 species). We provided one of the few quantitative overviews of Troidini egg survival and showed the negative effects of HR‐like cell death on egg survival rates. Additionally, we noted a large proportion of eggs oviposited in locations other than leaves, possibly suggesting a behavioral avoidance mechanism used by butterflies to escape egg‐killing by their host plants. Since our research was exploratory, sample sizes were dictated by the abundance and presence of butterflies. Future research would benefit from larger sample sizes and comparisons between *A. acuminata* plants with and without HR‐like responses in a comparative setting where multiple species are investigated in parallel. The addition of a temporal component, where the response is tracked over time, would provide valuable additional information. Overall, our results address a significant gap in the literature on the ecological interactions of Troidini eggs and *Aristolochia* and thereby provide essential ecological information with practical implications for conservation projects of these well‐known butterflies.

## Author Contributions

**Veerle van Beeck:** conceptualization (equal), data curation (lead), formal analysis (lead), funding acquisition (equal), investigation (equal), methodology (equal), project administration (equal), visualization (lead), writing – original draft (lead), writing – review and editing (equal). **Justinah Parantis:** investigation (supporting), methodology (supporting), resources (supporting), writing – review and editing (supporting). **Berber E. Zandstra:** data curation (supporting), investigation (supporting), writing – review and editing (supporting). **Tjomme Fernhout:** investigation (supporting), writing – review and editing (supporting). **Arthur Chung:** resources (supporting), writing – review and editing (supporting). **Nazirah Mustaffa:** resources (supporting), writing – review and editing (supporting). **Nina E. Fatouros:** conceptualization (equal), funding acquisition (equal), methodology (equal), resources (equal), supervision (equal), writing – review and editing (equal). **Corné F. H. van der Linden:** conceptualization (equal), funding acquisition (equal), investigation (equal), methodology (equal), project administration (equal), resources (equal), supervision (equal), writing – review and editing (equal).

## Funding

This work was supported by Uyttenboogaart‐Eliasen Stichting; Koninklijke Nederlandse Dierkundige Vereniging.

## Conflicts of Interest

The authors declare no conflicts of interest.

## Supporting information


**Data S1:** ece374426‐sup‐0001‐DataS1.xlsx.

## Data Availability

The data we based this research on is provided in the Supporting Information.
